# An Account of the Effects of Negative Electricity, in Cases of Burns

**Published:** 1797

**Authors:** John Vinall


XXII. *An Account of the Effects of Negative Electricity, in Cafes of Burns. By Mr.* John Vinall.—From the *Memoirs of the American Academy of Arts and Sciences,* Vol. II. Part I. 4to. Bofton, 1793.

IN making ufe of my large electrical machine, which is conftructed with both a pofitive, and a negative conductor, the air being humid, and confequently unfavourable for electrical experiments, I made ufe of a fmall iron pan with fome coals under the machine, in order to qualify the ſurrounding atmoſphere, ſo as to anſwer my purpoſe. By accident I burned my thumb with the pan ſo much, as to cauſe me great pain. Knowing that in ſome inſtances, I had been relieved of ſlight burns, by holding the part affected to a common fire, I held my thumb at a ſmall diſtance from the *negative* conductor; put the machine in motion; and to my ſurpriſe found, that in a few ſeconds of time, the effects of the burn were deſtroyed; that my thumb was perfectly at eaſe; and that no bliſter aroſe, as would, I think, have been the caſe if I had not made uſe of electricity. I met with a ſimilar inſtance not long after: I made uſe of the ſame remedy, and received the ſame benefit.

A few weeks after this diſcovery, one of my daughters ſcalded her arm from her wriſt to her elbow, with the ſteam from a tea kettle, which produced a great inflammation upon the part, attended with much pain; and it is highly probable a bliſter would have ſucceeded. I deſired her to hold her arm to the *negative* conductor; and in a few minutes, the pain ceaſed, the redneſs ſubſided, and her arm was perfectly cured.

I never read, or heard of an experiment of this kind being made in electricity: therefore, I eſteemed it my duty to lay it before the Academy demy of Arts and Sciences, that gentlemen might be induced to make experiments from this hint, which may be of great ſervice to mankind, and an improvement in medical electricity.

*Boſton, May* 23d,

1790.

